# Epidemiology of Migraines in Iraqi Females: Emphasis on Dietary Influence

**DOI:** 10.7759/cureus.44080

**Published:** 2023-08-24

**Authors:** Dina T Khafaf, Bilge Meral Koç

**Affiliations:** 1 Neurosciences, Bahcesehir University, Istanbul, TUR; 2 Nutrition and Dietetics, Bahçeşehir University, Istanbul, TUR

**Keywords:** iraq, epidemiology, dietary influence, females, migraines, neurological diseases

## Abstract

Introduction

Migraine is a neurological condition that frequently results in a severe headache. The headache comes in episodes and is occasionally accompanied by nausea, vomiting, and sensitivity to light. Migraines can be caused by a variety of conditions and can last anywhere from three to four hours to several days, with females experiencing them three times more frequently than men. Studies have found some evidence that lifestyle variables, such as nutrition, may play an important role in the emergence of migraines. The purpose of this research is to determine the epidemiology of migraine among females with an emphasis on the relationship between headaches and the dietary habits of females who are enduring migraine attacks in Iraq.

Methods

This study is descriptive research employing a quantitative method, specifically a survey. The data collection process involved a three-section online survey disseminated to females through internet platforms, including WhatsApp, Viber, Facebook, and Instagram. In this research, 360 females from Sulaymaniyah, Iraq, aged 18 to 35, participated. The survey's primary questions centered on the characteristics of the female respondents, drawing from the International Headache Society (IHS) criteria for migraine diagnosis. Participants meeting the migraine diagnostic criteria were also asked a few questions about aura symptoms. The Migraine Disability Assessment (MIDAS) questionnaire was incorporated, accompanied by inquiries about headache treatment, headache-related signs and symptoms, headache triggers, factors that relieve headaches, sleep routines, dietary consumption, and the impact of each factor on migraines.

Results

Of the 360 females who participated in the study, 159 (44.2%) experienced migraines, while 201 (55.8%) did not. The dietary habits of females who experienced migraines showed a statistically significant relationship to the duration of their headaches, specifically those lasting from 4 to 72 hours. This relationship was particularly evident in relation to nuts (p-value= 0.000), hot/spicy foods (p-value= 0.000), tomatoes (p-value= 0.005), bananas (p-value=0.01), aspartame (p-value=0.012), beverages containing caffeine (p-value=0.000), and citrusy fruits (p-value=0.008). These findings are based on p-values less than the commonly accepted alpha of 0.05. To maintain good health, it's essential to adhere to healthy eating habits and proper nutritional guidelines. Further research is necessary to identify additional dietary triggers for migraines. Enhancing data collection methods, such as using face-to-face interviews, could improve the quality of future research.

Conclusion

This study determined the prevalence of migraines among a sample of females in Sulaymaniyah, Iraq, and identified various foods consumed in excess by females without considering their potential impact on migraines.

## Introduction

Migraine is a common neurological condition, it is one of the most frequent types of headache disorders, and it can have a significant impact on a person's personal, professional, and social life. According to the WHO, severe migraine is one of the chronic conditions that have the potential to substantially impair one's ability to function. It is characterized by a strong and unbearable headache that is usually one-sided and pulsating. Other symptoms, including photophobia, nausea, vomiting, mood disturbances, dizziness, and abnormal sensory perceptions, sometimes accompany this type of headache. In women's health care, headaches are one of the most prevalent health disorders and a serious medical issue [1]. More than a billion people worldwide suffer from migraines yearly, making it one of the most common neurological conditions. The prevalence of migraines is particularly high in young individuals and females [2]. Of the entire adult population worldwide, 46% suffer from headaches, 11% from migraines, 42% from tension-type headaches, and 3% from chronic daily headaches. According to WHO, this would place headache disorders in the top ten most disabling conditions for both genders and the top five for females [3]. In order to accurately identify migraines, the International Headache Society (IHS) has established a set of diagnostic criteria. Firstly, a headache attack should persist for more than four consecutive hours and up to three days, with or without effective therapy. Moreover, the headache must exhibit at minimum two of the following properties: it must be unilateral, pulsating, severe or moderate intensity, or aggravate with regular exercise such as walking. Furthermore, headaches must appear with nausea, vomiting, photophobia (sensitivity to light), or phonophobia (fear of loud sounds). Lastly, the headache should not be attributed to a different disorder [4]. In general, medical students invest significant effort and are required to maintain a high level of focus and dedication throughout the day. Such rigorous commitment often leads to heightened stress, potentially resulting in sleep disturbances, especially given their exposure to highly stressful environments. A cross-sectional study was conducted among medical students in Jeddah, Saudi Arabia, over a one-year period. The participants displayed a headache and migraine prevalence notably higher than anticipated. Specifically, 26.3% reported migraines, with 41.6% of these individuals experiencing severe pain. Factors predicting migraines included the presence of Functional Gastrointestinal Disorders, a family history of migraines, being female, and enrollment in the second year of university. Stress and sleep disturbances emerged as the most common triggers. During migraine episodes, most participants reported challenges in attending educational sessions and a subsequent decline in academic performance [5].

Consequently, a person's likelihood of experiencing migraines and their ability to work and live better can be considerably increased by aspects contributing to their way of life. Neurologists frequently recommend that patients who suffer from migraines adopt a routine-based lifestyle. This is due to the observation that rapid shifts in any aspect of one's lifestyle, including dietary choices, can bring on migraine symptoms. Other aspects include physical activity, getting enough sleep, balancing work and rest, and so on [6]. Patients who experience headaches, particularly those who suffer from migraines, often indicate that dietary triggers cause their pain. Based on the study population and the methods used, the existence of any single food trigger in migraine patients ranges from 10% to 64%. Certain food types cause headaches in just an hour of intake, though others cause headaches 12 hours later. Previously, research has focused on identifying migraine triggers, including alcohol (specifically red wine), chocolate, caffeine, dairy products like aged cheese, food preservatives like nitrates and nitrites, monosodium glutamate, and sugar substitutes like aspartame [7]. The consumption of alcoholic beverages is commonly noted as a migraine trigger. Alcoholic beverages were found to be a trigger by 35.6% of migraine patients in a cross-sectional, web-based questionnaire survey [8]. Another prospective cross-sectional research showed that widely consumed dietary triggers were coffee (19.9%), chocolate (7.5%), and food rich in monosodium glutamate (5.6%) [9]. Surveys were conducted in a study with 171 participants, of which 132 were females and 39 were males. A total of 8.2% reported that aspartame was a definite migraine trigger [10]. As a serious disease, migraine is best characterized as having episodic appearances, progressing in some people, and having a significant socio-economic consequence. Migraine patients and their family members experience stressful situations due to the condition, affecting their ways of living and disrupting their daily interactions within the family. Since migraines are more prevalent in females, it is considered to be a significant health threat for them [1]. The purpose of this research is to determine the epidemiology of migraine among Iraqi females, emphasizing the relationship between headaches and the dietary habits of females enduring migraine attacks in Iraq.

## Materials and methods

The data collection process involved a three-section online survey that was sent to the females via internet platforms such as WhatsApp, Viber, Facebook, Instagram. When the survey was distributed to the females, an informed consent form was included. (Dear participants, I am sharing my Master's Degree research survey about the Epidemiology of Migraines in Females, with An Emphasis on Dietary Influence in Iraq. Participation is completely voluntary, and responses will be kept anonymous). To make the online survey easier for all the female respondents, it was written in a simplified form and was entirely in English. The survey's first section asked questions about the characteristics of the female respondents, including their age, marital status, etc. Next in the same section of the survey there were questions based on the (IHS) criteria for diagnosing migraines. This will distinguish between females who don't experience migraines and those who do. In addition, those who meet the migraine diagnostic criteria will also be asked a few questions about aura symptoms. The second section of the survey included an assessment of how much migraines influence the female's everyday life using a unique questionnaire called the Migraine Disability Assessment (MIDAS). The assessment results established the level of disability on a scale ranging from no or little disability to severe disability. A score from 0-5 indicated little or no disability, 6-10 indicates mild disability, 11-20 indicated moderate disability and more than 21 indicates severe disability. The total number of days should be added for all five questions to define the final disability level [11]. In the final section of the survey, questions regarding headache treatment, headache-related signs and symptoms, headache triggers, headache relieving factors, sleep routine, dietary consumption, and the influence of each factor on migraines was asked.

The type of study is descriptive research and that is a quantitative method (survey). The goal of descriptive research is to collect information that may be used to make statistical interpretation about a population. This requires careful planning and a well-structured design. In this research, 360 females participated from Sulaymaniyah, Iraq, with ages ranging from 18 to 35, all participating through different online platforms. By turning off the collect email addresses option in the survey's settings, the participants confidentiality and privacy were maintained.

An online survey was created using Google Forms for the data collection procedure. The survey was divided into three sections: The first section contained demographic questions, including age, marital status, occupation, weekly working hours, weight, and height, which were then used to determine BMI classifications. Within the same section, questions based on the International Headache Society (IHS) diagnostic criteria for migraines were included. Respondents who met the migraine diagnostic criteria were further probed about aura symptoms. The second section encompassed an evaluation of the impact of migraines on a female's daily life using the Migraine Disability Assessment (MIDAS) questionnaire. This assessment categorized the level of disability on a scale, ranging from little or no disability to severe disability. The final section posed questions about headache treatments, headache-related signs and symptoms, headache triggers, factors that alleviate headaches, sleep patterns, dietary habits, and the influence of each factor on migraines

The IBM SPSS Statistics version 24 program was utilized for data analysis. The data were coded, tabulated, and presented descriptively. The statistical procedures applied to ascertain the results of the current study included the Logistic Regression Model and Probability Value. For representing nominal data, frequencies and percentages were employed. The criteria for determining the significance of the test based on the p-value were as follows:

1. High significant (P<0.001)

2. Significant (P<0.05)

3. Non-significant (P>0.05)

4. Very highly significant (P<0.000)

The sample size was calculated according to a pre-established formula:

N = population size

e = Margin of error (percentage in decimal form)

Z = Z-score (desired confidence level, 95%: z score = 1.96)

The estimated sample size required to achieve a precision of ±0.05% with a 95% confidence interval (CI) was 450 females. Given an 80% response rate, 90 responses were excluded due to inaccurate data in the questionnaires. Consequently, a total of 360 respondents completed and returned the questionnaire via online platforms. A step-by-step data analysis is presented in the Findings section.

## Results

The participants of the study were exclusively female. Table 1 outlines their characteristics. Out of the total, 173 participants (48.1%) were aged between 18 and 26, while 187 (51.9%) were between 27 and 35 years old. Of these, 234 (65.0%) were single, and 126 (35.0%) were married. In terms of their profession, 218 (60.6%) were working, 61 (16.9%) were students, 50 (13.9%) balanced both studying and working, and 31 (8.6%) reported none. When questioned about their weekly work hours, 172 (47.8%) stated they worked more than 36 hours, 104 (28.9%) worked between 17 and 35 hours, 53 (14.7%) worked 16 hours, and 31 (8.6%) reported not working at all. Based on BMI calculations, 8 (2.2%) females were underweight, 204 (56.7%) had a normal weight, 118 (32.8%) were overweight, and 30 (8.4%) were classified as obese.

**Table 1:** Characteristics of the female participants.

Items	Frequency	%
Age		
18-26	173	48.1
27-35	187	51.9
Marital status		
Married	126	35.0
Single	234	65.0
Profession		
Student	61	16.9
Student + Working	50	13.9
Working	218	60.6
None	31	8.6
Working hours per week as student or employee		
16 hours per week	53	14.7
17-35 hours per week	104	28.9
More than 36 hours per week	172	47.8
None	31	8.6
BMI		
Under weight	8	2.2
Normal	204	56.7
Over weight	118	32.8
Obese	30	8.4
Total	360	100

Table 2 indicates that out of the 360 females who responded to the survey, 159 (44.2%) reported experiencing headaches that lasted between 4 and 72 hours. In contrast, 201 (55.8%) said they did not. Regarding headache characteristics based on the (IHS) criteria, respondents were presented with options such as experiencing pain on one side of the head, a pulsating sensation, moderate or severe pain intensity, aggravation of pain by daily activities (e.g., walking, climbing stairs, exercising) or the avoidance of these activities due to the pain, and symptoms of nausea and/or vomiting, as well as photophobia (sensitivity to light). The most frequently selected criterion was pain on one side of the head, reported by 111 (29.84%) respondents. Additionally, during headache episodes, 149 (68.98%) of the respondents experienced both photophobia (sensitivity to light sources such as computer screens or sunlight) and phonophobia (sensitivity to loud sounds).

**Table 2:** Distribution of headache frequencies and characteristics based on IHS criteria. IHS: International Headache Society.

Items	N=159	%
Do you have headaches lasting from 4 to 72 hours		
No	201	55.58
Yes	159	44.2
Total	360	100.0
Your headache characteristics (Select up to 4 answers based on your headaches)		
Moderate or severe pain	84	22.65
Pulsating (beating)	96	25.81
Pain in one side of your head	111	29.84
Pain increases by routine daily activity like walking, climbing stairs, exercise or you avoid doing these activities	81	21.77
Total	372	100.0
During headaches (Select up to 2 answers based on your headaches)		
Photophobia (sensitivity toward light, computer screen, sunlight) and phonophobia (sensitivity towards loud sounds)	149	68.98
Nausea and/or vomiting	67	31.02
Total	216	100.0

Table 3 presents the statistical analysis of the aura symptoms experienced by females and their characteristics in daily life. The most commonly reported aura symptom was 'motor aura (weakness in your body),' selected by 92 (30.98%) participants. The most frequently chosen aura characteristic was 'at least one aura symptom you selected previously spreads rapidly within 5 minutes,' reported by 51 (19.17%) participants.

**Table 3:** Distribution of frequency and percentages of aura symptoms and characteristics.

Items	N=159	%
Do you have any of these aura symptoms along with your headaches? (Select as much as you have)		
Speech and/or language (having difficulties speaking)	19	6.4
Motor aura (weakness in your body)	92	30.98
Brainstem (Ringing in ears, dizziness)	60	20.20
Sensory aura (numbing or tingling in the limbs "hands or feet")	39	13.13
Visual aura (seeing sparks or flashes, not seeing well, any other similar symptoms including visual lose)	77	25.93
None	10	3.37
Total	297	100
Characteristics of these aura symptoms in your daily life? (Select as much as you have)		
At least one aura symptom you selected previously rapidly spreads throughout 5 minutes	51	19.17
At least one symptom of aura is unilateral (occur on one side of your body)	46	17.29
The presence of at least one positive aura symptom (Seeing flashes or sparks)	47	17.67
Two or more aura symptoms happen one after the other	26	9.77
Headache comes with the aura or comes after it within 60 minutes	48	18.05
Each aura symptom lasts between 5 and 60 minutes	38	14.29
None	10	3.76
Total	266	100

The results for the MIDAS questionnaire are as follows: There are four score types. A score of 0-5 days indicates little or no disability, 6-10 days indicates mild disability, 11-20 days indicates moderate disability, and over 21 days indicates severe disability. The total number of days was added across all five questions to determine the final disability level. The statistical data was handled manually, and the answers to the questions were aggregated. The results show that 93 participants (58.49%) scored between 0 and 5, indicating little or no disability; 31 participants (19.49%) scored between 6 and 10, indicating mild disability; 24 participants (15.0%) scored 11-20, indicating moderate disability; and 11 participants (6.9%) scored more than 21, indicating severe disability. Table 4 illustrates the effects of medication on headaches. Out of the respondents, 97 (61.0%) reported a positive effect, 6 (3.8%) reported no effect, and 56 (35.2%) did not respond. Regarding relief after taking medication, 78 (49.1%) reported relief within 1-2 hours, 14 (8.8%) within 3-5 hours, 6 (3.8%) within 6-12 hours, 5 (3.1%) after more than a day, and 56 (35.2%) did not specify. Without medication, 8 respondents (5.0%) said their headache lasted 1-2 hours, 27 (17.0%) for 3-5 hours, 38 (23.9%) for 6-12 hours, 30 (18.9%) for more than a day, and 56 (35.2%) did not specify. Regarding the signs and symptoms of a headache, photophobia (sensitivity to light) was the most commonly reported symptom with 107 respondents (30.57%), while diarrhea was the least common with 6 respondents (1.71%).

**Table 4:** Distribution of frequency and percentages of medication benefit, medication functioning hours, headache duration, and headache signs and symptoms.

Items	N=159	%
Do you find that taking medicine helps relieve your headaches in any way?		
No	6	3.8
Yes	97	61.0
None	56	35.2
How long does it often take for your headaches to calm after taking your medication?		
1-2 Hours	78	49.1
3-5 Hours	14	8.8
6-12 Hours	6	3.8
More than a day	5	3.1
None	56	35.2
Without medications, what is the duration of your headaches?		
1-2 Hours	8	5.0
3-5 Hours	27	17.0
6-12 Hours	38	23.9
More than a day	30	18.9
None	56	35.2
Which Headache Related Sign and Symptoms Do you experience? (You can select few or all answers based on the symptoms that you have).		
Feeling cold	30	8.57
Dizziness	90	25.71
Nausea/Vomiting	64	18.28
Photophobia (Not tolerating Light)	107	30.57
Sweating	26	7.42
Diarrhea	6	1.71
Other	27	7.71
Total	350	100

Table 5 shows the results for the possible headache triggers. Stress is identified as the most common factor that may trigger headaches, with a percentage of 115 (12.9%).

**Table 5:** Distribution of frequency and percentages of headache triggers.

Items	N=159	%
Physical activity (exercise, daily activities)	68	7.6
Skipping meals (not eating)	85	9.5
Loud noises	80	9.0
Stress	115	12.9
Sleep disturbance	114	12.8
Feeling sad	89	10.0
Lights/Sunlight	86	9.6
Hot weather	64	7.2
Certain smells (Perfumes...etc.)	50	5.6
Menstrual cycle (menstruation)	90	10.1
Certain food types	47	5.2
Total	888	100

The results for possible headache-relieving factors are displayed in Table 6. Resting is identified as the most common factor that may relieve headaches, with a percentage of 121 (22.0%).

**Table 6:** Distribution of frequency and percentages of headache relievers.

Which factors do you feel relieves your headache? (You can select a few or all answers based on your experience).	N=159	%
Drinking caffeinated drinks (Coffee, Coca-Cola)	45	8.1
Resting	121	22.0
Sleeping	117	21.3
Sitting in a quite room with no lights	73	13.2
Medications	98	17.8
Messages	56	10.2
Eating	27	4.9
Vomiting	12	2.1
Total	549	100

**Figure 1:**
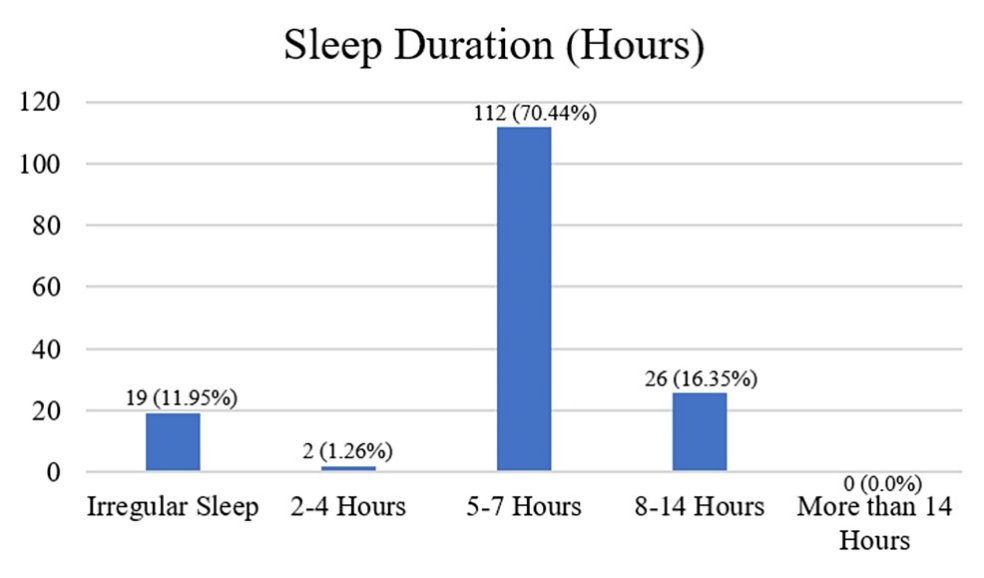
Vertical bar graph showing number and percentages of sleeping hours.

**Figure 2:**
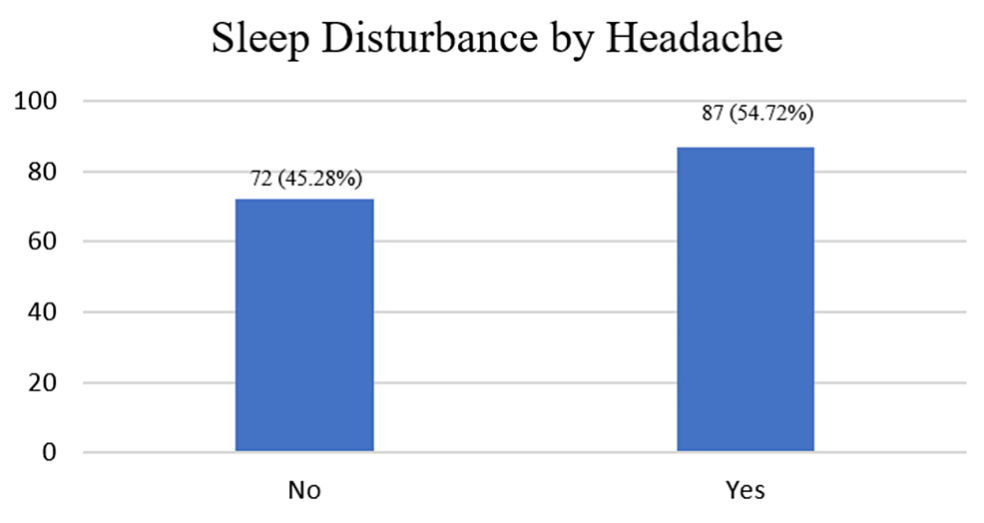
Vertical bar graph showing the number and percentages of participants whose sleep is disrupted by headaches.

The results detailing the sleeping hours of female participants, as well as their responses to whether headaches disturb their sleep, are displayed in Figures 1 and 2. The breakdown for sleeping hours is as follows: 2 (1.26%) reported sleeping for 2-4 hours, 112 (70.44%) for 5-7 hours, 26 (16.35%) for 8-14 hours, 19 (11.95%) had irregular sleep patterns, and 0 (0.0%) slept for more than 14 hours. Of the participants, 87 (54.72%) answered 'yes' to headaches disturbing their sleep, while 72 (45.28%) answered 'no'.

Table 7 displays the participants' dietary consumption patterns and the subsequent changes they noticed in their headaches. Of the participants, 119 (74.84%) reported consuming chocolate, and 36 (22.64%) of them noticed a change in their headache after consumption. Similarly, 128 (80.50%) participants consumed cheese, with 38 (23.90%) observing a change in their headache afterward. Nuts like almonds and peanuts were consumed by 134 (84.28%) of the participants, and 62 (38.99%) reported a change in their headache after consumption. Additionally, 99 (62.26%) participants said they drink milk, and 41 (25.79%) observed a headache change post-consumption. When it came to hot or spicy foods, 96 (60.38%) participants consumed them, with 32 (20.13%) noting a subsequent headache change. Lastly, 144 (90.57%) of the respondents consumed tomatoes, and 39 (45.53%) reported a change in their headache after doing so. Out of the participants, 127 (79.87%) responded affirmatively to consuming bananas, while 32 (20.12%) responded negatively. Of them, 60 (37.74%) noticed a change in their headaches after consuming bananas. Similarly, 127 (79.87%) of the participants consumed yogurt, with 32 (20.13%) declining. A total of 44 (27.67%) reported a change in their headaches to post-yogurt consumption. As for aspartame, 39 (24.53%) consumed it, while 120 (75.47%) did not, with 14 (8.81%) noting a headache change after consumption. When considering beverages with caffeine like coffee, tea, or coke, 148 (93.08%) of the participants consumed them, while 11 (6.92%) did not. 45 (28.30%) observed a change in their headaches after consumption. Regarding citrus fruits such as lemons, oranges, and grapefruits, 126 (79.25%) participants consumed them, and 33 (20.75%) did not; 31 (19.50%) reported a headache change after consumption. For smoked fish, 54 (33.96%) of the participants consumed it, while 105 (66.04%) did not. After consumption, 23 (14.47%) reported a change in their headaches.

**Table 7:** Distribution of frequency and percentages of participants' responses to dietary consumption.

Items		Food consumption (N=159)		Consumption time (N=159)			Change after consumption (N=159)			
		No	Yes	Daily	2-4 per week	None	Not sure	No difference	Yes	
Chocolate	Fr.	40	119	43	76	40	40	83	36	
	%	25.16	74.84	27.04	47.80	25.16	25.16	52.20	22.64	
Cheese	Fr.	31	128	34	94	31	31	90	38	
	%	19.50	80.50	21.38	59.12	19.50	19.50	56.60	23.90	
Nuts: Almonds, Peanuts. etc.	Fr.	25	134	22	112	25	25	72	62	
	%	15.72	84.28	13.84	70.44	15.72	15.72	45.28	38.99	
Milk	Fr.	60	99	24	75	60	60	58	41	
	%	37.74	62.26	15.09	47.17	37.74	37.74	36.48	25.79	
Hot/Spicy foods	Fr.	63	96	24	72	63	63	64	32	
	%	39.62	60.38	15.09	45.28	39.62	39.62	40.25	20.13	
Tomato	Fr.	15	144	70	74	15	15	105	39	
	%	9.43	90.57	44.03	46.54	9.43	9.43	66.04	45.53	
Banana	Fr.	32	127	18	109	32	32	67	60	
	%	20.12	79.87	11.32	68.55	20.12	20.12	42.14	37.74	
Citrusy Fruits such as Lemons, Oranges and Grapefruit or drink their Juices	Fr.	33	126	19	107	33	33	95	31	
	%	20.75	79.25	11.95	67.30	20.75	20.75	59.75	19.50	
Smoked Fish	Fr.	105	54	1	53	105	105	31	23	
	%	66.04	33.96	0.63	33.33	66.04	66.04	19.50	14.47	
Yoghurt	Fr.	32	127	35	92	32	32	83	44	
	%	20.13	79.87	22.01	57.86	20.13	20.13	52.20	27.67	
Aspartame (an artificial sweetener and sugar substitute) as part of your daily dietary intake	Fr.	120	39	20	19	120	120	25	14	
	%	75.47	24.53	12.58	11.95	75.47	75.47	15.72	8.81	
Beverages containing caffeine, such as coffee, tea, or coke	Fr.	11	148	131	17	11	11	103	45	
	%	6.92	93.08	82.39	10.69	6.92	6.92	64.78	28.30	
I drink, (You can select up to 3 as many as you drink)	Items	Coffee		Tea		Coke		Total		
	Fr.(%)	107 (38.22)		125(44.64)		48(17.14)		280 (100)		

Table 8 presents the relationship between headaches lasting from 4 to 72 hours and participants' responses to dietary consumption, as analyzed by the logistic regression model. The results indicate a statistically significant association between headaches lasting from 4 to 72 hours and the consumption of nuts (p-value= 0.000), hot/spicy foods (p-value= 0.000), tomatoes (p-value= 0.005), bananas (p-value=0.01), aspartame (p-value=0.012), beverages containing caffeine (p-value=0.000), and citrusy fruits (p-value=0.008). This significance is determined by a p-value less than the standard alpha level of 0.05. Conversely, there appears to be no statistical significance between headaches of the same duration and the consumption of chocolate (p-value=0.501), cheese (p-value= 0.424), milk (p-value=0.913), yogurt (p-value=0.684), and smoked fish (p-value=0.06) as their p-values exceed the standard alpha level of 0.05.

**Table 8:** Relationship between headaches lasting from 4 to 72 hours and participants' response to dietary consumption using the logistic regression model.

Items	Exp (B)	95% CI for Exp (B)		Significance
		Lower	Upper	
Chocolate	1.218	0.685	2.167	0.501
Cheese	1.281	0.698	2.35	0.424
Nuts	3.319	1.846	5.967	0.000
Milk	1.032	0.588	1.81	0.913
Hot/Spicy foods	2.646	1.545	4.529	0.000
Tomato	2.833	1.366	5.873	0.005
Banana	2.01	1.183	3.417	0.01
Yoghurt	1.139	0.608	2.134	0.684
Aspartame	1.954	1.157	3.3	0.012
Beverages containing caffeine	5.071	2.701	9.521	0.000
Citrusy Fruits	2.296	1.246	4.232	0.008
Smoked Fish	0.564	0.311	1.024	0.06

## Discussion

In this study, female participants from Sulaymaniyah, Iraq, shared their experiences with migraines. Of the 360 females who participated, 159 (44.2%) reported experiencing migraines, while 201 (55.8%) did not. Regarding the dietary habits of those who experienced migraines, it was found that certain foods or drinks exacerbated their headaches. The most commonly consumed item was caffeinated beverages (coffee, tea, coke) with 148 (93.08%) reporting consumption, followed by tomatoes at 144 (90.57%), nuts at 134 (84.28%), cheese at 128 (80.50%), bananas and yogurt each at 127 (79.87%), citrusy fruits at 126 (79.25%), chocolate at 119 (74.84%), milk at 99 (62.26%), spicy foods at 96 (60.38%), smoked fish at 54 (33.96%), and aspartame at 39 (24.53%). The relationship between the duration of headaches (ranging from 4 to 72 hours) and participants' responses to dietary consumption was analyzed using a logistic regression model. Results revealed a statistically significant association between headaches lasting from 4 to 72 hours and the consumption of nuts (p-value= 0.000), hot/spicy foods (p-value= 0.000), tomatoes (p-value= 0.005), bananas (p-value=0.01), aspartame (p-value=0.012), caffeinated beverages (p-value=0.000), and citrusy fruits (p-value=0.008). This significance was determined because the p-values were less than the standard alpha level of 0.05. Conversely, no statistical significance was observed between such headaches and the consumption of chocolate (p-value=0.501), cheese (p-value= 0.424), milk (p-value=0.913), yogurt (p-value=0.684), and smoked fish (p-value=0.06), as their p-values exceeded 0.05. When examining potential headache triggers and relievers, stress was identified as the most common trigger, reported by 115 (12.9%) of participants. Conversely, resting was recognized as the most effective reliever, chosen by 121 (22.0%) of the participants. Results from a study on gender differences and migraine prevalence in Baghdad City, Iraq, which involved 230 migraine patients, revealed that females are more prone to migraines than males. Of the participants, 138 (60%) were females and 92 (40%) were males [12]. In contrast, our study exclusively surveyed 360 female participants, of whom 159 (or 44.2% of the total) reported experiencing migraines. The findings are relatively in line with the earlier study from Baghdad. A separate cross-sectional study conducted on secondary school students in Ramadi, Iraq, aimed to determine the prevalence of migraine headaches. Out of a total of 1445 students, 94 (6.5%) were diagnosed with migraines. The number of female students diagnosed was higher than that of male students, with figures of 59 (8.9%) for females and 35 (4.9%) for males [13]. This prevalence is notably different from the results of our study. This discrepancy might be attributed to the overall lower number of migraine diagnoses in the Ramadi study. It's also important to note the inclusion of both genders in their research, in contrast to our all-female cohort. The results of a study examining the patterns of migraine headaches in a group of Kurdish Iraqi patients in Erbil, Iraq, revealed that a majority of the participants suffering from migraines were females. Of the 200 participants, 153 (or 76.5%) were females and 47 (or 23.5%) were males [14]. In contrast, our research exclusively surveyed 360 female participants, while the Erbil study included both genders. A separate study on the triggers and remedies for headaches was conducted among 200 Iraqi Kurdish patients in Erbil. Stress or psychological upset was identified as the most common trigger for migraines in 80% of participants, followed by heightened physical activity in 68%. When it came to alleviating migraines, nonsteroidal anti-inflammatory drugs (NSAIDs) were effective for 50% of the patients, while sleep proved beneficial for 45.5%. In our research, stress was also found to be a prominent migraine trigger, but at a different prevalence of 115 participants (or 12.2%) [15]. However, our findings diverged regarding migraine relief; in our study, resting was the most commonly cited remedy, reported by 121 participants (or 22.0%). According to a review article, the pathogenesis of migraines and headaches is thought to be influenced by dietary compounds. Various foods have been discovered to be related to migraine headaches. Following the available research, common foods linked to migraines include chocolate, citrus fruits, ice cream, onions, dairy products, nuts, alcoholic beverages, coffee, caffeine, histamine, tyramine, monosodium glutamate (MSG), phenylethylamine, aspartame, sucralose, nitrates, and gluten [16]. Additionally, when consumed in large quantities, certain foods and ingredients have been discovered to cause migraines. Particularly, the reaction to an episode of headache is primarily triggered by the quantity and timing of the food. Furthermore, research shows that certain food and food component triggers may further cause headaches in patient subgroups who suffer from other medical conditions and immunological reactions to food, such as positive IgG antibodies. These patients are more likely to experience headaches due to the triggers. The identification of dietary triggers is made significantly more difficult by these health problems. In specific patients, migraine dietary triggers are identified through serological testing and food diaries [16]. In a systematic literature review about the role of diet and nutrition in migraine triggers and treatment, it has been explained that the association of dietary patterns with migraine has also been reported in a number of cross-sectional studies and surveys. As an example of a cross-sectional study in the United States, people who experience migraines with aura were found to be more likely to have a low intake of chocolate (p-value = 0.005), cheese (p-value = 0.008), ice cream (p-value = 0.003), hot dogs (p-value < 0.001), and processed meats (p-value = 0.009), in comparison to those who experience migraines without aura [17]. In our study, we explored the association between various food items and headaches, specifically evaluating the relationship between dietary intake and headaches that last between 4 and 72 hours in females, both with and without aura. Our results indicate a statistically significant association between the duration of headaches (from 4 to 72 hours) and the consumption of certain foods: nuts (p-value= 0.000), hot/spicy foods (p-value= 0.000), tomatoes (p-value= 0.005), bananas (p-value=0.01), aspartame (p-value=0.012), beverages containing caffeine (p-value=0.000), and citrus fruits (p-value=0.008). Furthermore, in a study from Jeddah, Saudi Arabia, which included only female participants, chocolate and caffeinated beverages were identified as migraine triggers. Approximately 10% of the respondents noted a correlation between migraine onset and the consumption of chocolate and caffeine. Specifically, 7.8% of participants identified caffeine as the primary trigger for their headaches, while only 2.2% attributed it to chocolate [18]. In comparison, among our sample of 159 female participants, caffeinated beverages (such as coffee, tea, and coke) were predominant, with 148 (93.08%) reporting consumption. The Departments of Child Neurology and Child Psychiatry at Ankara University conducted a cross-sectional study involving school-aged children. The aim was to investigate the relationship between childhood headaches and various factors, including leisure time activities, depression, anxiety, and eating habits. The study comprised a total of 5355 school-aged students from 12 schools. An analysis of the results pertaining to nutritional habits revealed that out of the 550 students with migraines, 252 (45.8%) reported that their migraine episodes were triggered by food consumption. Specifically, 108 (19.6%) identified cola and coffee, 50 (9.1%) pointed to fried food, 17 (3.1%) to spicy food, 16 (2.9%) to chocolate, 15 (2.7%) to canned products, 13 (2.4%) to tea, 13 (2.4%) to peanuts, 12 (2.2%) to cheese, and eight students (1.5%) mentioned other foods such as sausages, salami, fish, and fruit as migraine triggers [19]. Some of these dietary items correspond with our findings, as our study also indicated a significant association between migraines and the consumption of caffeine-containing beverages, spicy foods, and nuts, each with a p-value of 0.000.

Recommendations

More research should be done regarding the prevalence of migraine in the female population of Iraq. It is crucial to conduct more research with a larger sample of female participants to learn more about how each dietary item affects migraine occurrence. Healthy eating and a correct nutrition style must be followed in order to maintain healthy eating habits. Therefore, in Iraq, public awareness of the correlation between migraines and dietary habits should be introduced, and education should be provided in schools, universities, and workplaces. Also, more research is needed to identify new dietary migraine triggers. Finally, data collection improvements, such as face-to-face interviews, may improve research quality.

## Conclusions

The aim of this study was to gather data regarding the frequency of migraines among a sample of Iraqi females who participated in the survey and to understand the relationship between their most frequently consumed foods and the onset of migraines. Potential migraine-triggering foods for some individuals include caffeine (found in coffee, tea, and colas), spicy foods, nuts, aspartame, and certain fruits and vegetables such as tomatoes, bananas, lemons, and oranges. Females are still experiencing migraines at high rates, which has a negative impact on both standards of living and performance. Migraine can trigger depression, stress, and anxiety disorders. It can also result in stroke and other heart diseases, such as coronary heart disease and high blood pressure. This study highlights the importance of being aware of the potential effects of certain foods on migraines, especially among females.
